# Newborn and infant hearing health education for nursing professionals

**DOI:** 10.5935/1808-8694.20130039

**Published:** 2015-11-02

**Authors:** Camila Padilha Barbosa, Juliana Barbosa Aires, Isabela Yasmin dos Santos Farias, Francisca Márcia Pereira Linhares, Silvana Maria Sobral Griz

**Affiliations:** a MSc in Nursing - Federal University of Pernambuco - UFPE. Speech and Hearing Therapist; b Nursing Student - Federal University of Pernambuco - UFPE; c PhD in Nutriton - Federal University of Pernambuco - UFPE. Professor at the Department of Nursing - UFPE; d PhD in Cognitve Psychology - Federal University of Pernambuco - UFPE. Professor at the Graduate Program in Human Communicaton Health

**Keywords:** health education, hearing, nursing

## Abstract

**Abstract:**

Hearing loss is a sensory deprivation, which can brings several consequences, among them: language development delay, emotional and social problems, and school difficulties. In such settings, we stress the role of nursing professional, who can expand their knowledge about children's hearing health, all the way from pre-natal care.

**Objective:**

To check the knowledge of nursing professional after the educational actions on pediatric hearing.

**Method:**

A quasi-experimental design with time-series outline. We had 82 nursing professional participating (nurses, technicians and assistants) working in a university hospital between March and September of 2011. All of the interviewees answered a semi-structured questionnaire before and after the educational actions.

**Results:**

We observed a significant change in the knowledge of the nursing professionals after the educational activity in most of the variables; such as: ideal age to perform the newborn hearing screening; ideal age to diagnose hearing loss; ideal age to start intervention against hearing loss and risk indicators for hearing loss.

**Conclusion:**

It is believed that the methodology used in educational activities, based on problems found in professional practice, education may have contributed greatly to increase knowledge about hearing health, especially concerning neonates and infants.

## INTRODUCTION

Hearing is characterized as a fundamental condition for oral language development. Therefore, it is closely related to human communicaiton^1^. Hearing loss is a sensorial deprivation and, among its consequences, we may list: language development delays; cognitive, social/emotional and school performance problems^2^. This makes hearing loss be considered as a public health problem, requiring greater attention from society, from the government and from healthcare professionals^1^, because late diagnosis may bring about losses which impact on the child's global development^3^.

Hearing health promotion is the first step of a Children Auditory Health Program (CAHP) which must be started already in pre-natal care. One important stage in these programs is the Neonatal Hearing Screening (NHS), which allows for the detection of possible auditory changes in neonates and infants^4^. Not least important are the health educational actions which may expand the knowledge of healthcare professionals and users on the topic. Such actions aim at contributing to the identification of auditory changes as soon as possible, as well as for the intervention at the ideal time, in other words, by the sixth month of life^4^, ^5^.

In these settings, we highlight nursing professionals, who may act as multipliers of knowledge on children auditory health^6^. For such, these professionals must learn through continuing learning processes and be ready to assist and provide the population with full health support by means of health education actions^7^.

Paulo Freire's methodology proposes the conceptualization of educational actions from its practice^8^, in order to change it, improving it, correcting mistakes, or even completely changing certain practices^9^. In these initiatives, activities are proposed to involve the continuous process of education, enabling the instructor to perceive reality and change it, given its relationship with the environment^10^.

Since there is a need for greater disclosure about children auditory health and be able to work with the theme, based on Paulo Freire's problematic, this study aimed at checking the knowledge of nursing professionals after educational programs on children auditory health.

## METHOD

This is a quasi-experimental study with a temporal-series outline. We had 82 nursing professionals (RNs, technicians and assistants) participating from a university hospital, who worked in the puericulture, pediatrics, neonatal intensive care wards, maternity and obstetric center, between March and September of 2011. The inclusion criterion to participate in the study was to be present in at least three of the meetings proposed for the educational initiatives.

Data collection was carried out at three moments: (1) Initial questionnaire deployment **-** in this stage, the Nursing Professionals answered a semi-structured questionnaire; (2) Educational Programs - Based on the information collected in the questionnaire, we developed themes approached in the educational actions based on the precepts of Paulo Freire's methodology, which core is associated with the problematic as a starting point^11^. We had four meetings to discuss the topic of pediatric auditory health, focusing on neonates and infants, led by a speech and hearing therapist. These meetings lasted for one hour on average, and were held every 30 days, in average. The content was presented as slide projections, video presentations, group dynamics and rounds of conversation, approached as problematic, analyzed and interpreted, resulting on the creation/recreation of knowledge, in a dialogical relationship between the researcher and the nursing professional. Based on what was approached in meetings 1, 2 and 3, we developed an educational folder, distributed to the participants in the last meeting, representing an educational support material for them, besides enabling the nursing professional to use it in their practice and (3) Questionnaire Redeployment - this stage was carried out at the end of the fourth meeting, when the same semi-structured questionnaire was redeployed.

The data was entered in a database, in the *Statistical Package for the Social Sciences* (SPSS) software, version 13.0. In data analysis we calculated the percentage frequencies for the variables studied and we built the contingency tables of interest. In order to assess the numerous factors studied, we used the Chi-square test for independence. On the tables in which the Chi-square test assumptions were not met, we used the Fisher's exact test. To assess the professionals' levels of knowledge before and after the educational programs, we used the Chi-square test for homogeneity; and we used the Chi-square test for proportion in comparing the percentages of simple frequency distributions. In all the conclusions we considered a significance level of *p* ≤ 0.05. This study was approved by the Ethics in Research Committee, under protocol number 0130.0.172.000-10.

## RESULTS

Among the nursing professionals who participated in this study (n = 82), 80 were females and two were males, with ages between 21 and 63 years (mean of 41.6 years). As far as education is concerned, 52.4% (n = 43) had higher education, with graduation time and professional carrier time of less than 20 years - 58.5% (n = 48) and 56.1% (n = 46), respectively. Most nursing professionals (84.1%, n = 69) stated they did not receive information concerning auditory health during their professional training.

We stress that: (a) 35.4% (n = 29) worked as a nurse, (b) 26.8% (n = 22) of the nursing professionals worked in the Neonatal Intensive Care Unit (NICU) and (c) 97.6% (n = 80) worked only in a public hospital (Table 1).

**Table 1:** Demographic characteristics of nursing professionals. Recife, 2011.

Demographic variables	n	%
Hospital role		
Nurse	29	35.4
Nurse technician	28	34.1
Nurse assistant	25	30.5
Hospital department		
NICU	22	26.8
Obstetrical center	19	23.2
Pediatrics	18	22.6
Joint quarters	17	20.7
Residents	6	7.3
Workplace		
Public hospital only	80	97.6
Public and private hospital	2	2.4
Total	82	100.0

We can observe a change in the knowledge of nursing professionals after the educational program in different variables. In regards of the knowledge of nursing professionals as to the need to assess the hearing of neonates and infants with/without a hearing loss risk indicator (HLRI) and as to the knowledge about which hospitals of the region do the NHS (neonatal hearing screening), we noticed that there had been no significant change after the educational program. It was not possible to do the significance statistical test for the analysis of the knowledge of the nurses on the need to assess the hearing of neonates and infants with HLRI, since they all reported that it was relevant to investigate the hearing of this population, before and after the educational initiative (Table 2).

**Table 2:** Nursing professionals' knowledge before and after the educational program. Recife, 2011.

Knowledge about	Before the program		After the program		Total		***p***-value
	n	%	n	%	n	%	
Hearing assessment without HLRI							
Yes	78	95.1	80	97.6	158	96.3	0.681^2^
No	4	4.9	2	2.4	6	3.7	
Hearing assessment with HLRI							
Yes	82	100.0	82	100.0	164	100.0	-
No	0	-	0	-	0	-	
Ideal age to perform NHS							
Yes	63	76.8	79	96.3	142	86.6	< 0.001^1^,*
No	19	23.2	3	3.7	22	13.4	
Ideal age to diagnose the hearing loss							
Yes	18	22.0	57	69.5	75	45.7	< 0.001^1^,*
No	64	78.0	25	30.5	89	54.2	
Ideal age to start the intervention							
Yes	15	18.3	59	71.9	74	45.1	< 0.001^1^,*
No	67	81.7	23	28.1	90	54.8	
Hearing loss consequences							
Yes	72	87.8	81	98.8	153	93.3	0.004^2^
No	10	12.2	1	1.2	11	6.7	
Hearing loss prevalence							
Yes	0	-	60	73.2	60	36.6	< 0.001^1^,*
No	82	100.0	22	26.8	104	63.4	
Hearing loss types							
Yes	1	1.2	74	90.2	75	45.7	< 0.001^1^,*
No	81	98.8	8	9.8	89	54.3	
Hearing loss levels							
Yes	4	4.9	71	86.6	75	45.7	< 0.001^1^,*
No	78	95.1	11	13.4	89	54.2	
Hearing loss risk indicators							
Yes	53	64.6	81	98.8	134	81.7	< 0.001^1^,*
No	29	35.4	1	1.2	30	18.2	
Socioeconomic and demographic factors							
Yes	71	86.6	80	97.6	151	92.1	0.009^1^
No	11	13.4	2	2.4	13	7.9	
Hearing assessment tests							
Yes	69	84.1	82	100.0	151	92.1	< 0.001^1^,*
No	13	15.9	0	-	13	7.9	
Professionals who do the NHS							
Yes	70	85.4	82	100.0	152	92.7	< 0.001^1^,*
No	12	14.6	0	0.0	12	7.3	
Hospitals that do the NHS							
Yes	78	95.1	82	100.0	160	97.5	0.120^2^
No	4	4.9	0	0.0	4	2.4	
Total	82	100.0	82	100.0	164	100.0	-

^1^ *p*-value from the Chi-square test for homogeneity

^2^ *p*-value from the Fisher's exact test

^*^ Statistically significant values (*p* ≤ 0.05).

Most nursing professionals reported that the first month of life is ideal to do the NHS, before (76.8%, n = 63) and after (96.3%, n = 79) the educational program (Table 3).

**Table 3:** Knowledge of nursing professional about: ideal age to perform the Neonatal Hearing Screening, hearing loss diagnosis and intervention, before and after the educational program. Recife, 2011.

Knowledge	Before the educational program		After the educational program		Total		***p***-value
	n	%	n	%	n	%	
Ideal age to be submitted to Neonatal Hearing Screening							
By the first month	64	78.1	79	96.3	143	87.2	
By the third month	12	14.6	3	3.7	15	9.2	
By the sixth month	4	4.9	0	-	4	2.4	0.001^1^
From 1 year	1	1.2	0	-	1	0.6	
Does not know	1	1.2	0	-	1	0.6	
Ideal age to diagnose the hearing loss							
By the first month	27	32.9	17	20.7	44	26.9	
By the third month	18	22.0	57	69.5	75	45.7	
By the sixth month	17	20.7	6	7.3	23	14.0	< 0.001^1^
From 1 year	19	23.7	2	2.4	21	12.8	
Does not know	1	1.2	0	-	1	0.6	
Ideal age to start intervention							
By the first month	14	17.1	4	4.9	18	11.0	
By the third month	7	8.5	9	11.0	16	9.8	
By the sixth month	15	18.3	68	82.9	83	50.6	< 0.001^1^
From 1 year	43	52.4	1	1.2	44	26.8	
Does not know	3	3.7	0		3	1.8	
Total	82	100.0	82	100.0	164	100.0	

^1^ Statistically significant values (*p* ≤ 0.05) - Fisher's exact test.

On Table 4, we notice a significant increase on the answers regarding the tests which assess hearing: transient otoacoustic emissions (TOE) of 59.8% (n = 49) to 96.3% (n = 79); tonal audiometry of 46.3% (n = 38) to 86.6% (n = 71); Brainstem Auditory Evoked Potential (BAEP) of 3.7% (n = 3) to 64.6% (n = 53) and immittance measuring of 1.2% (n = 1) to 26.8% (n = 22).

**Table 4:** Nursing professional knowledge on the tests which help in hearing assessment, before and after the educational program. Recife, 2011.

Knowledge	Before the educational program		After the educational program		Total		*p*-value
	n	%	n	%	n	%	
Neonatal Hearing Screening							
Yes	49	59.8	79	96.3	128	78.0	
No	20	24.4	3	3.7	23	14.0	< 0.001^1^
Does not know	13	15.8	0	-	13	8.0	
Tonal audiometry							
Yes	38	46.3	71	86.6	109	66.5	
No	31	37.8	11	13.4	42	25.6	< 0.001^1^
Does not know	13	15.8	0	-	13	8.0	
BAEP							
Yes	3	3.7	53	64.6	56	34.1	
No	66	80.5	29	35.4	95	57.9	< 0.001^1^
Does not know	13	15.8	0	-	13	8.0	
Immittance measuring							
Yes	1	1.2	22	26.8	23	14.0	
No	68	82.9	60	73.2	128	78.0	< 0.001^1^
Does not know	13	15.8	0	-	13	8.0	
Total	82	100.0	82	100.0	164	100.0	

^1^ Statistically significant values (*p* ≤ 0.05) - Chi-squared test for homogeneity.

Concerning the conduct of such professional regarding neonates and infants under risk for hearing loss, there was a difference on the answers regarding: the need to refer neonates and infants to NHS of 28.0% (n = 23) to

35.4% (n = 29); refer to a specialist: from 20.7% (n = 17) to 14.6% (n = 12); not knowing what to do (17.1%, n = 14) to educate the parents concerning NHS (29.3%, n = 24).

## DISCUSSION

The attitudes and influence of nursing professionals on a given population is impressive^12^. This makes them need to be educated on specific themes, through educational programs which make a problematic of its practice. Such programs empower them with knowledge coming from other areas, such as hearing health^13^.

This study's sample was made up mainly of females. This is a profession's trait which reflects socio-historical aspects^14^, since nursing came up simultaneously to home-care of children, elderly and sick people, associated with a woman-mother figure who played the role of a healer, having informal knowledge on health practices, passed on from woman to woman. Male nurses take on more administrative and political roles than healthcare roles^15^ and, when working with healthcare, they prefer emergency or intensive care, places with technological progresses and appreciation for a multiprofessional teamwork.

Although 52.4% of the participants in this study had higher education - 64.6% worked in middle-level roles - as nursing technicians or assistants. It is common for nursing professionals to start their professional lives in middle level programs, even with the possibility of moving on to higher education^16^. The growth of university-based nursing programs^17^ has also contributed to this.

Thus, children auditory health themes must be added to the training of nurses, as we consider the attitudes and influence of nursing professionals on the people they work with^12^. These professionals may help in the development of NHS programs. Notwithstanding, most of the participants (84.1%) reported they did not receive information on hearing health during their training, even when 58.5% had concluded their training in the past 20 years - a time when recommendations on children hearing health, especially that of neonates and infants has intensified^4^, ^18^.

Despite the fact that all nursing professionals who work with pregnant women, parturients, neonates and infants need knowledge on children auditory health^19^, we stress the participation of those who worked in the NICU, because of the high incidence of hearing loss in neonates who spend time at the NICU^1^.

Among the relevant aspects associated with children hearing health, we know that UNHS is recommended since 1994^18^, and this is a first step in the assessment, diagnosis and intervention when there is hearing loss. Ideally, it is recommended that the NHS should be carried out by the first month of life, the diagnosis by the third month of life and intervention must start by the sixth month of life^4^, ^5^. The results from this study have shown that educational programs were positive, changing the level of knowledge of the professionals. As to the NHS, most of the participants (78.1%) reported it was ideal by the first month of life, even before the educational programs. Their knowledge expanded after they participated in the educational meetings (96.3%). The partnership between the nursing professionals and the NHS service may favor the effectiveness of these programs, since nursing is a *habitué* profession in other types of neonatal screenings, fostering the increase in coverage and the number of exams^20^. We stress that the work of nurses, in general, is closer to mothers and neonates, in their hospital stay, being able to provide parents with information and procedures to do the NHS^21^.

As to the ideal age to perform the audiological diagnosis and the ideal age to start intervention, we noticed that, in the beginning, the interviewees did not know when the ideal time for intervention was. This lack of knowledge may lead to a late diagnosis of hearing loss and, consequently, late intervention. Thus, there is no question about the need for information. Even before the educational programs most of the interviewees reported knowing about the consequences of hearing loss.

It is estimated that neonatal hearing loss prevalence is approximately 1 to 6:1,000 births, being higher than in newborns coming from the NICU^1^. Despite this fact, we noticed that before the educational program, 100% of the professional who worked in the NICU stated they were unaware of such information. This result was changed at the end of the educational program, when 73.2% of the nursing professionals were aware of the hearing loss prevalence in the general population and in the population who were under HLRI. This knowledge may expand and change conducts with the neonate, as per approached in another study^22^.

Knowledge on the type and degree of hearing loss is also convenient in order to associate such factors to the etiology of hearing loss and its consequences. Although this knowledge is specific for the professionals who work with hearing, this aspect enables healthcare professionals to have a different look on children who bear certain etiologies associated with hearing loss with specific levels and types. As a result, the expectation is to minimize the losses in oral language development^23^.

The lack of information on children hearing health during the education of nursing professionals and the separation between teaching and practice may corroborate the lack of knowledge about HLRI^19^. In this study, we observed that over 60% (64.6%) of the nursing team knew about some HLRI. After the educational program, this knowledge was expanded, being mentioned by 98.8% of the participants. However, even before the educational program, most (95.1%) agreed that it was relevant to investigate hearing independently from the presence of risk indicators, and such knowledge changed to 97.6% after the educational program.

Other risk indicators that may impact health are socioeconomic and demographics. Since the definition of health is broad^24^, to consider such aspects is fundamental to assess the risks to which a population is exposed^25^. Before the educational program, nursing professionals had a broad understanding of health, mentioning some socioeconomic and demographic aspects as influential in healthcare. Thus, more attention must be given to these issues associated with NHS-results^26^.

NHS involves TOAE and/or BAEP^27^. Other tests are part of the necessary battery of tests used in audiological diagnosis. In the beginning of this study, 95.1% of the professionals stated knowing about NHS, but did not include it among the tests in the audiological exams battery. After the educational program, all the participants knew at least one of the tests used in the diagnosis. This change in knowledge may cause the nursing professionals to work in partnership with others from a PSAI team, especially the hearing and speech therapist, in regards of the ideal protocol for a given population^28^. Besides knowing of the specific tests used in auditory detection and diagnosis, it is also important that the professionals be aware of which are the specialized services and to whom they must refer the families to^23^.

At the end of this study, all the professionals who participated in the program could properly educate the families, strengthening the multidisciplinary work, and making it so that all the recommended stages for identification and intervention in the first months of life are complied with^29^. Thus the importance of educational programs or continuing education, in order to foster better prognosis for pediatric auditory health.

This study may guide on the creation of continuing educational programs among healthcare professional concerning the auditory health of neonates and infants, aiming at intervention, started as soon as possible, thus minimizing the consequences of hearing loss on the individual's life and that of his/her family and the State.

In time, we suggest a future study with the redeployment of the questionnaire, six months after the end of the educational programs to check the knowledge acquired and added to practice. The setting up of a Family Counseling service may also be an interesting initiative, providing information and education, supported by hearing and speech therapists, since they are the healthcare professionals able to act in these full efforts to identify hearing loss as soon as possible.

## CONCLUSION

Educational programs on the hearing health of neonates and infants have significantly changed the knowledge of nursing professionals. The methodology utilized in the educational programs, based on problematizing daily life, may contribute to the increase of knowledge on the theme.
